# Identifying optimal candidates for postoperative adjuvant therapy among regional persistent/recurrent nasopharyngeal carcinoma patients after neck dissection

**DOI:** 10.1186/s12885-022-10150-0

**Published:** 2022-11-03

**Authors:** Sai-Lan Liu, Xiao-Yun Li, Xue-Song Sun, Jing-Yun Peng, Chao Lin, Jin-Jie Yan, Qiu-Yan Chen, Lin-Quan Tang, Shan-Shan Guo, Ling Guo, Li-Ting Liu, Hai-Qiang Mai

**Affiliations:** 1 Sun Yat-sen University Cancer Center; State Key Laboratory of Oncology in South China; Collaborative Innovation Center for Cancer Medicine, Guangdong Key Laboratory of Nasopharyngeal Carcinoma Diagnosis and Therapy, 651 Dongfeng Road East, Guangzhou, 510060 People’s Republic of China; 2 Department of Nasopharyngeal Carcinoma, Sun Yat-Sen University Cancer Center, 651 Dongfeng Road East, Guangzhou, 510060 People’s Republic of China

**Keywords:** Regional recurrent nasopharyngeal carcinoma, Neck dissection, Postoperative adjuvant therapy, Plasma Epstein–Barr virus, Prognosis

## Abstract

**Purpose:**

To analyze the clinical outcomes of patients with regional persistent/recurrent nasopharyngeal carcinoma (NPC) who received neck dissection, and to evaluate the clinical benefit of postoperative adjuvant therapy (PAT) based on patients’ positive lymph node counts (PLNs), extracapsular spread (ECS) and preoperative plasma EBV DNA levels.

**Methods:**

From 2003 to 2017, 342 patients with regional persistent/recurrent NPC were included in this study. All patients were treated with neck dissection and 76 patients received PAT. Progression-free survival (PFS), overall survival (OS), distant metastasis-free survival (DMFS) and locoregional relapse-free survival (LRFS) were compared between groups using propensity score matching (PSM).

**Results:**

152 patients without PAT treatment and 76 patients with PAT treatment were selected by the PSM. There was no significant difference in 2-year PFS (52.4% vs. 61.3%, *P* = 0.371), 2-year OS (91.9% vs. 90.5%, *P* = 0.097) or 2-year LRFS (66.3% vs. 67.9%, *P* = 0.872) between the two groups. However, the application of PAT brought survival benefits to patients in terms of 2-year DMFS (76.5% vs. 84.7%, *P* = 0.020). PLN, ECS and preoperative EBV DNA level remained independent risk factors for poorer PFS. Accordingly, patients were divided into low-risk and high-risk groups using receiver operating characteristic (ROC) curve; the 2-year PFS rates for two risk groups were 73.4% and 59.1% (*P* < 0.0001) respectively. The results showed that low-risk patients didn’t benefit from the addition of PAT. However, the 2-year DMFS rate was significantly improved in high-risk PAT-treated patients than those treated by neck dissection alone (83.7% vs. 71.7%, *P* = 0.023).

**Conclusions:**

PLNs, ECS and preoperative EBV DNA level are associated with the prognosis of patients with regional persistent/recurrent NPC. High-risk patients identified by PLNs, ECS and preoperative EBV DNA level may benefit from the addition of PAT after neck dissection.

**Supplementary Information:**

The online version contains supplementary material available at 10.1186/s12885-022-10150-0.

## Introduction

Nasopharyngeal carcinoma (NPC) is a unique malignant cancer arising from the nasopharynx, and concomitant Epstein-barr virus (EBV) infection was observed in most cases [1, 2]. 70–80% of NPC patients present with lymph node metastasis at diagnosis [3]. With the development of modern imaging and radiation techniques, such as intensity-modulated radiotherapy (IMRT), nodal metastasis is usually eradicated after primary chemoradiotherapy. However, there are still 4% to 18% of persistent or recurrent nodal diseases after definitive chemoradiotherapy [4–8]. Patients with regional failure can still be salvaged with additional therapy, and long-term survival is achievable. According to the National Comprehensive Cancer Network (NCCN) guidelines, radical or modified neck dissection (ND) with or without radiotherapy is recommended as the primary salvage treatment for NPC patients solely with regional failure. Meanwhile, various postoperative strategies including brachytherapy, external beam radiotherapy and chemotherapy have been employed. Due to the extent of invasion of tumor, great difference in surgical type and subsequent adjuvant therapies, the 5-year overall survival (OS) of patients varied from 25 to 70%, with the 5-year regional control rate from 60 to 80% [9–12]. In addition, the role of postoperative adjuvant therapy (PAT) is uncertain, from which not all patients may benefit. Therefore, it requires further investigation on the role of PAT following salvage ND, along with the appropriate candidates for the administration of PAT.

Therefore, we conducted this retrospective study to compare the survival outcomes of regional persistent/recurrent NPC patients with or without adjuvant therapy after ND, and to identify the group of patients who may benefit from PAT.

## Methods

### Patients

Patients who underwent neck dissection for persistent/recurrent nodal disease between January 2003 and December 2017 in Sun Yat-sen University Cancer Center (SYSUCC) were identified. Persistent disease is diagnosed when the metastatic lymph node continued to exist 3 months after the radical radiotherapy, whereas recurrent disease is defined as the reappearance of metastatic lymph nodes after initial complete recession. The inclusion criteria were as followed: [1] aged 18–70 years; [2] biopsy-proven World Health Organization type II or III NPC before the primary treatment; [3] pathologically confirmed persistent/recurrent nodal disease; [4] received previous radical radiotherapy; [5] absence of local recurrence, distant metastasis, secondary malignancy, pregnancy or lactation. This study was approved by the Ethics Committee of SYSUCC, and because of the observational nature of this study, the requirement for informed consent was waived.

### Treatment

Radical neck dissection (RND) or selective neck dissection (SND) was administered in all patients. SND was implemented in patients with isolated metastatic node or recurrence identified in less than 3 consecutive levels through imaging tests or intraoperative findings. Otherwise, RND was performed.

Based on the results of intraoperative exploration and postoperative histopathological data, postoperative adjuvant treatment was conducted in a certain proportion of patients, such as those with extranodal invasion. For postoperative radiation, accumulated doses of 50 to 60 Gy in 25 to 30 fractions were administered to the neck region by IMRT. The chemotherapy regimen concurrently with reirradiation was cisplatin in 80 or 100 mg/m^2^ per cycle for 2–3 cycles. Common postoperative chemotherapy regimens consist of the following: TPF: docetaxel (60 ~ 75 mg/m^2^, day 1) or paclitaxel (135 mg/m^2^, day 1), cisplatin (60 ~ 75 mg/m^2^, day 1) and 5-fluorouracil (3 ~ 3.75 g/m^2^ civ120h); PF: cisplatin (80 mg/m^2^, day 1) in combination with 5-fluorouracil (4 g/m^2^ civ120h); and GP: gemcitabine (1 g/m^2^, day 1/ 8) plus cisplatin (80 mg/m^2^, day 1) for 3 or 4 cycles. The treatment regimens were chosen according to patients’ past treatment history and clinician’s judgment.

### Data collection and analysis

We collected demographics and clinical information of included patients, such as recurrent N (rN) stage, preoperative plasma EBV DNA level, positive lymph node counts (PLNs), the state of extracapsular spread (ECS) according to postoperative pathology and surgical methods, etc. ECS was defined as the invasion of neoplastic cells into perinodal fibrillar connective tissue or adipose tissue under microscope. Results were interpreted independently by two experienced pathologists. According to preoperative imaging examination, rN stage was restaged using the 8th Edition of the Union for International Cancer Control (UICC) TNM staging system. Quantitative polymerase chain reaction was used to measure the preoperative plasma EBV DNA levels of patients as described in a previous study [13]. The preoperative plasma EBV DNA level was divided into detectable and undetectable (cut-off value: 0 copy/mL) [14, 15]. Patients who received postoperative treatment, including chemotherapy alone, radiotherapy alone or chemoradiotherapy, were grouped into ND + PAT group. For further comparisons, every patient in ND + PAT group was matched to two patients in the ND alone group according to propensity scores calculated by the covariates: age, gender, persistent or recurrent disease, rN stage, preoperative plasma EBV DNA level, PLNs, ECS, maximal diameters of LNs, bilaterality and surgical methods. The cut-off value was selected for each clinicopathological factor according to the median or results from previous studies.

### Outcome and follow up

The primary endpoint for the study was progression-free survival (PFS), defined as the time from the initial date of treatment for persistent/recurrent nodal disease to date of death, or treatment failure at any site. Patients who didn’t experience any event were censored at the date of the last follow-up. The secondary endpoints included OS (defined as the time to date of death from any cause), locoregional relapse-free survival (LRFS, defined as the time to date of local/regional relapse), and distant metastasis-free survival (DMFS, defined as the time to the date of distant metastasis). After the completion of treatment, patients were examined every 3 months during the first 3 years and every 6 months thereafter or until death. Nasopharyngoscopy, enhanced MRI of the head and neck, chest radiography, abdominal sonography, or PET-CT were routinely performed at every follow-up visit or upon clinical indication of tumor recurrence.

### Statistical analysis

Statistical analyses were generated with R software (http://www.R-project.org, 4.0.2). χ2 test (or Fisher’s exact test if indicated) was used to assess categorical variables, whereas the t-test and Mann–Whitney U test were used to analyze continuous variables. To reduce the potential confounders caused by selection bias, the propensity score matching (PSM) method was performed. The actuarial survival rates and survival curves were estimated by the Kaplan–Meier method and compared using the log-rank test. Associations between potential covariates and outcomes were analyzed using the Cox proportional hazards models, and the Hazard ratios (HRs) with 95% confidence intervals (CIs) were calculated. A *p* value less than 0.05 (two-tailed) was considered statistically significant.

## Results

### Patient characteristics

From 2003–2017, 342 patients were involved in this study. There were 266 (77.8%) patients treated by ND alone, and 76 (22.2%) treated by ND plus PAT. Among 76 patients treated with PAT, 18 (23.7%) patients received postoperative radiotherapy; 44 (57.9%) patients received chemotherapy and 14 (18.4%) patients received chemoradiotherapy. The detailed demographic and clinicopathologic features of patients (Table 1) were presented in the ND group and ND + PAT group, respectively. Compared with regional recurrent patients, a higher proportion of patients with nodal residual accepted ND alone (*P* = 0.036). Patients presenting with advanced rN stage (N3) (36.8% vs. 17.7%; *P* = 0.002), more PLNs (55.3% vs. 35.3%; *P* = 0.002), or ECS (51.3% vs. 34.2%; *P* = 0.007) were more inclined to accept PAT after dissection. Additionally, patients in the ND + PAT group were associated with higher preoperative EBV DNA level than patients in the ND alone group, but to a near-significant extent (*P* = 0.072). Other variables were comparable between the two groups. After PSM with a ratio of 1:2, a well-balanced cohort of 228 patients remained in the analysis, with 76 from the ND + PAT group and 152 from the ND alone group. The median age was 45 (18–70) years old, including 45 (19.7%) females and 183 (80.3%) males. No statistically significant differences in potential prognostic factors were observed in these two groups. The details of patients’ characteristics were shown in Table 1.

**Table 1:** Difference in patients’ characteristics between the ND alone group and ND + PAT group in the original observational and propensity-matched cohorts

	Observational dataset (*n* = 342)			PSM dataset(*n* = 228)		
**Characteristic**	ND	ND + PAT	*P*	ND	ND + PAT	*P*
**Total**	266	76		152	76	
**Age, y**			0.954			0.925
< 45	141(53.0)	40(52.6)		81(53.3)	40(52.6)	
≥ 45	125(47.0)	36(47.4)		71 (46.7)	36(47.4)	
**Gender**			0.159			0.724
Female	70(26.3)	14(18.4)		31(20.4)	14(18.4)	
Male	196(73.7)	62(81.6)		121(79.6)	62(81.6)	
**Status of lymph node**			0.036			0.120
Recurrence	201(75.6)	66(86.8)		119(78.3)	66(86.8)	
Residual	65(24.4)	10(13.2)		33(21.7)	10(13.2)	
rN stage*			0.002			0.538
N1	205(77.1)	46(60.5)		102(68.9)	46(60.5)	
N2	14(5.3)	2(2.6)		5(3.3)	2(2.6)	
N3	47(17.7)	28(36.8)		45(29.6)	28(36.8)	
**Preoperative EBV DNA level**			0.072			0.554
undetectable	129(48.5)	28(36.8)		50(32.9)	28(36.8)	
detectable	137(51.5)	48(63.2)		102(67.1)	48(63.2)	
**PLNs**			0.002			0.261
≤ 2	172(64.7)	34(44.7)		80(52.6)	34(44.7)	
> 2	94(35.3)	42(55.3)		72(47.4)	42(55.3)	
**Maximal diameter of LNs (mm)**			0.555			0.651
> 20	72(27.1)	18(23.7)		32(21.1)	18(23.7)	
≤ 20	194(72.9)	58(76.3)		120(78.9)	58(76.3)	
**ECS**			0.007			0.399
Yes	91(34.2)	39(51.3)		69(45.4)	39(51.3)	
No	175(65.8)	37(48.7)		83(54.6)	37(48.7)	
**Bilaterality**			0.132			0.435
Unilateral	256(96.2)	70(92.1)		144(94.7)	70(92.1)	
Bilateral	10(3.8)	6(7.9)		8(5.3)	6(7.9)	
**Surgical methods**		0.616		1.000		
SND	210(78.9)	62(81.6)		124(81.6)	62(81.6)	
RND	56(21.1)	14(18.4)		28(18.4)	14(18.4)	
**postoperative adjuvant therapy**			NA			NA
Radiation	NA	18 (23.7)		NA	18 (23.7)	
Chemotherapy	NA	44 (57.9)		NA	44 (57.9)	
Chemoradiotherapy NA		14 (18.4)		NA	14 (18.4)	

*Abbreviations*: *EBV* Epstein–Barr virus, *PLN* positive lymph node, *ECS* extracapsular spread, *RND* radical neck dissection, *SND* selective neck dissection

^*^According to the 8^th^ edition of UICC/AJCC staging system

### Survival outcomes

**Fig. 1:**
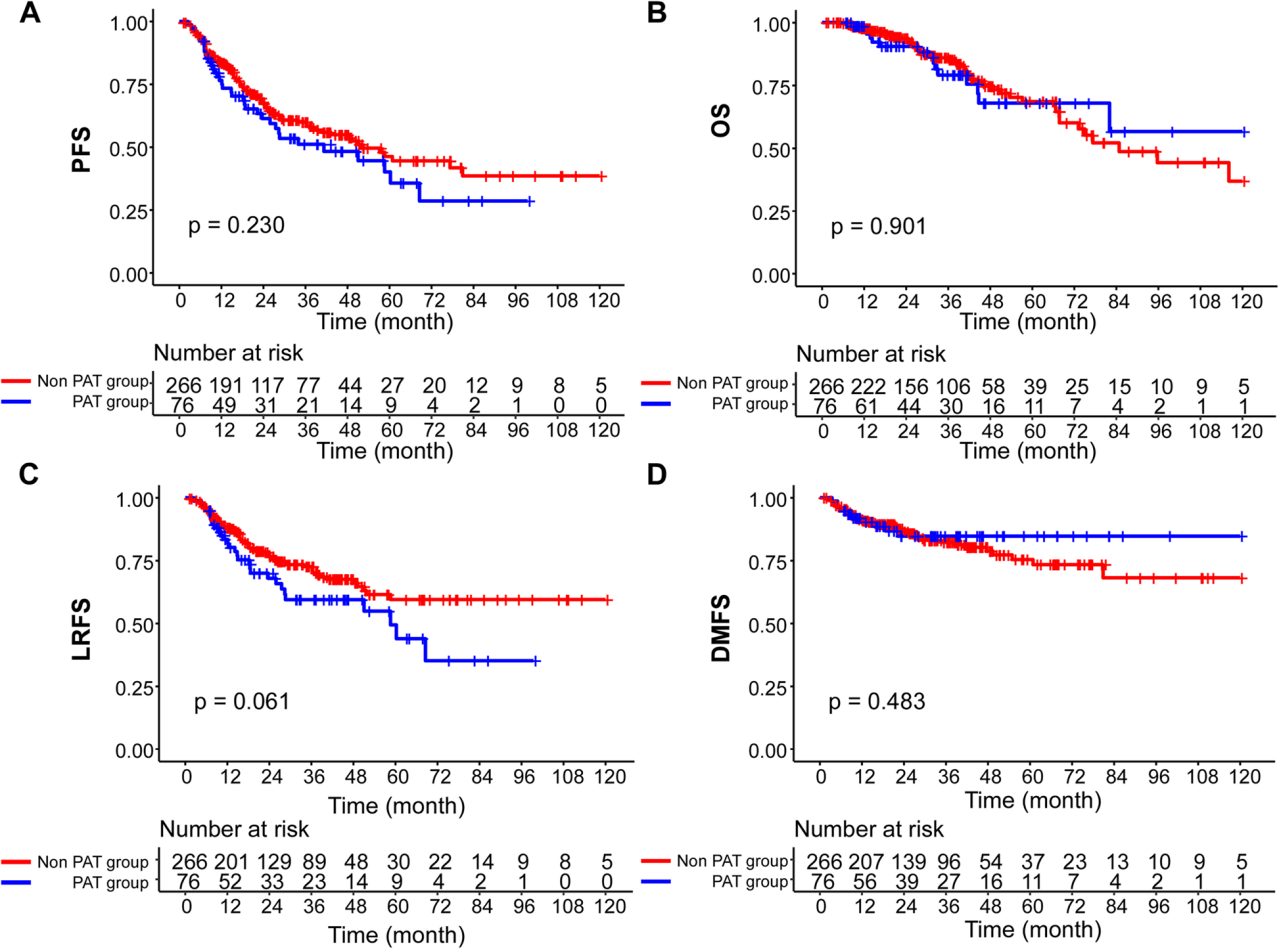
Kaplan–Meier curves for (**A**) progression-free survival (PFS), **B** overall survival (OS), **C** locoregional relapse-free survival (LRFS), and (**D**) distant metastasis-free survival (DMFS) in the original cohort of 342 patients with regional persistent/recurrent NPC

In the original cohort of 342 patients, the median follow-up time was 28.8 months in the ND alone group and 31.3 months in the ND + PAT group respectively. Overall, there was no significant difference in 2-year rates of PFS, OS, LRFS and DMFS between the two groups. (PFS: 67.3% vs. 61.3%, *P* = 0.230; OS: 93.6% vs. 90.5%, *P* = 0.901; LRFS: 76.5% vs. 67.9%, *P* = 0.061 and DMFS: 86.5% vs. 84.7%, *P* = 0.483, Fig. 1A-D). In multivariate analysis, the following variables were incorporated in the Cox proportional hazards model: age (y) (> 45 vs. ≤ 45); sex (male vs. female); recurrent N stage (1–2 vs. 3); preoperative EBV DNA level (detectable vs. undetectable); PLNs (> 2 vs. ≤ 2); ECS (yes vs. no); surgical methods (SND vs. RND); bilaterality (unilateral vs. bilateral); status of lymph node (recurrent vs. residual); maximal diameter of LNs (> 20 mm vs. ≤ 20 mm); type of treatment (ND + PAT vs. ND alone). As shown in Table 2, PLNs (HR, 1.488; 95% CI, 1.060–2.087; *P* = 0.021), ECS (HR, 1.908; 95% CI, 1.363–2.671; *P* < 0.0001) and preoperative EBV DNA level (HR, 1.686; 95% CI, 1.184–2.401; *P* = 0.004) remained as independent risk factors for poorer PFS. In addition, ECS was shown to be the independent risk factor for all other survival outcomes, including OS, LRFS and DMFS. ND combining adjuvant therapy failed to bring survival benefits in multivariable analysis in terms of PFS, OS, DMFS and LRFS in the whole cohort (all *p* values were > 0.05).

**Table 2:** Summary of the multivariable analyses of prognostic factors in original cohort

**Characteristic**	B	Hazard ratio (95% CI)	*P*
**Progression-free survival**			
preoperative EBV DNA level	0.522	1.686(1.184–2.401)	0.004
PLNs	0.397	1.488(1.060–2.087)	0.021
ECS	0.646	1.908(1.363–2.671)	< 0.001
**Overall survival**			
PLNs	0.892	2.441(1.460–4.081)	0.001
ECS	1.331	3.787(2.243–6.393)	< 0.001
Status of lymph node	0.742	2.099(1.203–3.662)	0.009
**Loco-regional relapse-free survival**			
preoperative EBV DNA level	0.668	1.950(1.277–2.979)	0.002
ECS	0.651	1.918(1.290–2.852)	0.001
**Distant metastasis-free survival**			
PLNs	0.565	1.760(1.015–3.050)	0.044
ECS	0.810	2.249 (1.307–3.869)	0.003
Surgical methods	0.825	2.281(1.023–5.085)	0.044

*Abbreviations*: *CI* confidence interval

HRs and *p* values were calculated using an adjusted multivariate Cox proportional hazards regression model, Age (y) (> 45 vs. ≤ 45); Sex (M vs. F); rN stage (1–2 vs. 3); EBV DNA (> 0 vs. 0 copy/ml); PLNs (> 2 vs. ≤ 2); ECS (yes vs. no); surgical methods (SND vs. RND); Bilaterality (unilateral vs. bilateral); Status of lymph node (Recurrence vs. Residual); Maximal diameter of LNs (> 20 mm vs. ≤ 20 mm); Type of treatment (surgery with postoperative treatment vs. surgery alone) were included as covariates. Variables were selected with the backward stepwise approach, and the p value threshold was 0.1 (*p* > 0.1) for removing insignificant variables from the model. Only variables significantly associated with survival were presented, and marginally significant variables (0.05 < *p* < 0.1) were remained in the final Cox model but not presented in the table

*EBV* Epstein–Barr virus, *PLN* positive lymph node, *ECS* extracapsular spread, *RND* radical neck dissection, *SND* selective neck dissection

**Fig. 2:**
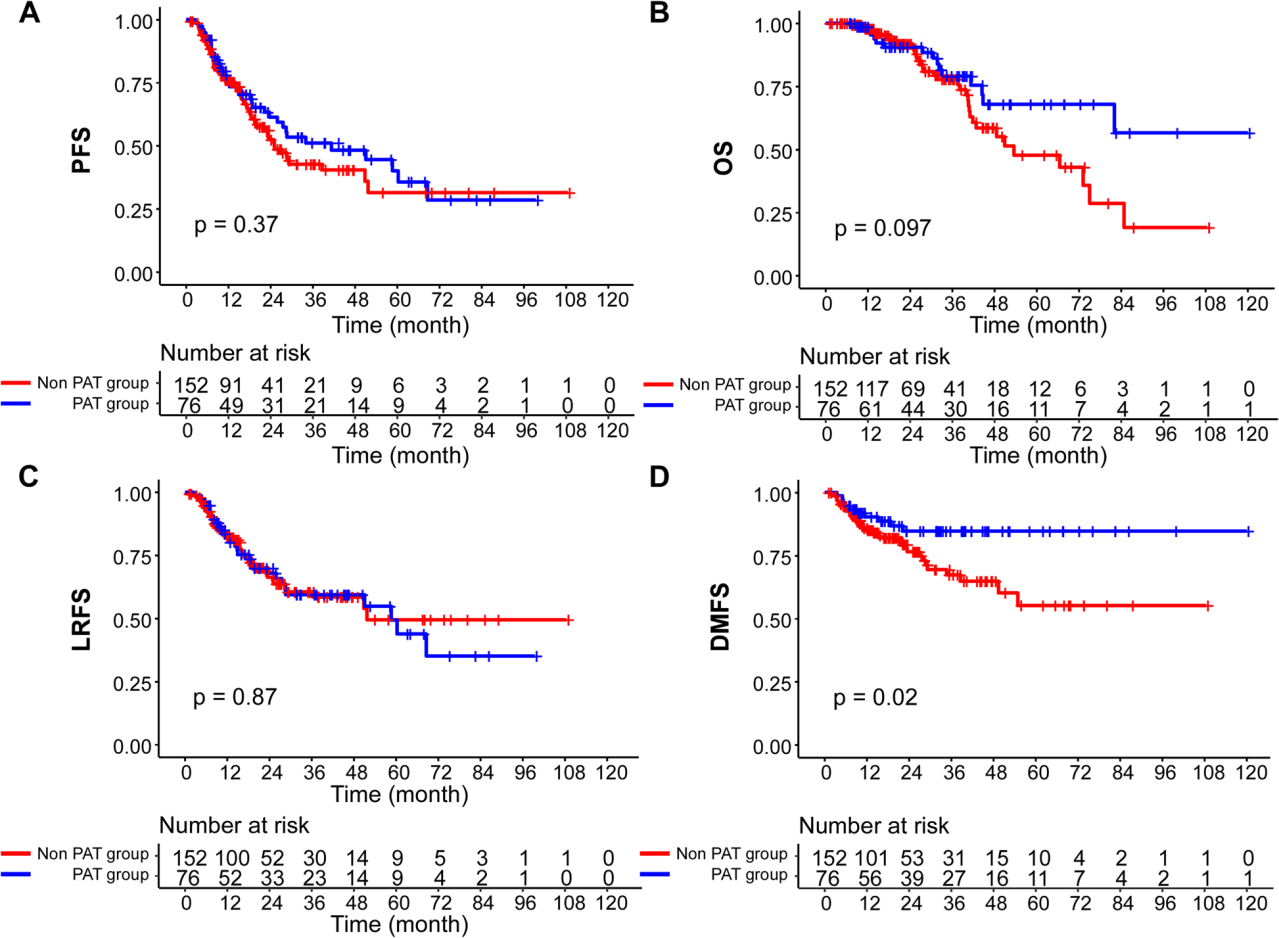
Kaplan–Meier curves for (**A**) progression-free survival (PFS), **B** overall survival (OS), **C** locoregional relapse-free survival (LRFS), and (**D**) distant metastasis-free survival (DMFS) in the PSM cohort of 228 patients with regional persistent/recurrent NPC

In the PSM cohort of 228 patients, the median follow-up time was 21.6 months in the ND alone group and 31.3 months in the ND + PAT group respectively. The application of adjuvant therapy following ND resulted in parallel PFS, OS and LRFS to ND alone group (2-year PFS: 52.4% vs. 61.3%, *P* = 0.371; OS: 91.9% vs. 90.5%, *P* = 0.097 and LRFS: 66.3% vs. 67.9%, *P* = 0.872, Fig. 2A-C). However, the survival improvement in DMFS was observed in the ND + PAT group (76.5% vs. 84.7%, *P* = 0.020, Fig. 2D), and the improvement was maintained in the multivariable analysis (HR, 0.662; 95% CI, 0.465–0.941; *P* = 0.021; Table 3).

**Table 3:** Summary of the multivariable analyses of prognostic factors in PSM cohort

**Characteristic**	B	Hazard ratio (95% CI)	*P*
**Progression-free survival**			
ECS	0.602	1.826(1.223–2.727)	0.003
**Overall survival**			
ECS	1.333	3.793(2.016–7.136)	< 0.001
Status of lymph node	0.900	3.293(1.794–6.044)	< 0.001
**Loco-regional relapse-free survival**			
ECS	0.565	1.760(1.113–2.784)	0.016
**Distant metastasis-free survival**			
Type of treatment	-0.413	0.662(0.465–0.941)	0.021
ECS	0.603	1.827(1.010–3.305)	0.046

*Abbreviations*: *CI* confidence interval

HRs and *p* values were calculated using an adjusted multivariate Cox proportional hazards regression model, Age (y) (> 45 vs. ≤ 45); Sex (M vs. F); rN stage (1–2 vs. 3); EBV DNA (> 0 vs. 0 copy/ml); PLNs (> 2 vs. ≤ 2); ECS (yes vs. no); surgical methods (SND vs. RND); Bilaterality (unilateral vs. bilateral); Status of lymph node (Recurrence vs. Residual); Maximal diameter of LNs (> 20 mm vs. ≤ 20 mm); Type of treatment (surgery with postoperative treatment vs. surgery alone) were included as covariates. Variables were selected with the backward stepwise approach, and the p value threshold was 0.1 (*p* > 0.1) for removing insignificant variables from the model. Only variables significantly associated with survival were presented, and marginally significant variables (0.05 < *p* < 0.1) were remained in the final Cox model but not presented in the table

*EBV* Epstein–Barr virus, *PLN* positive lymph node, *ECS* extracapsular spread, *RND* radical neck dissection, *SND* selective neck dissection

### Risk stratification according to PLNs, ECS and preoperative EBV DNA level

**Fig. 3:**
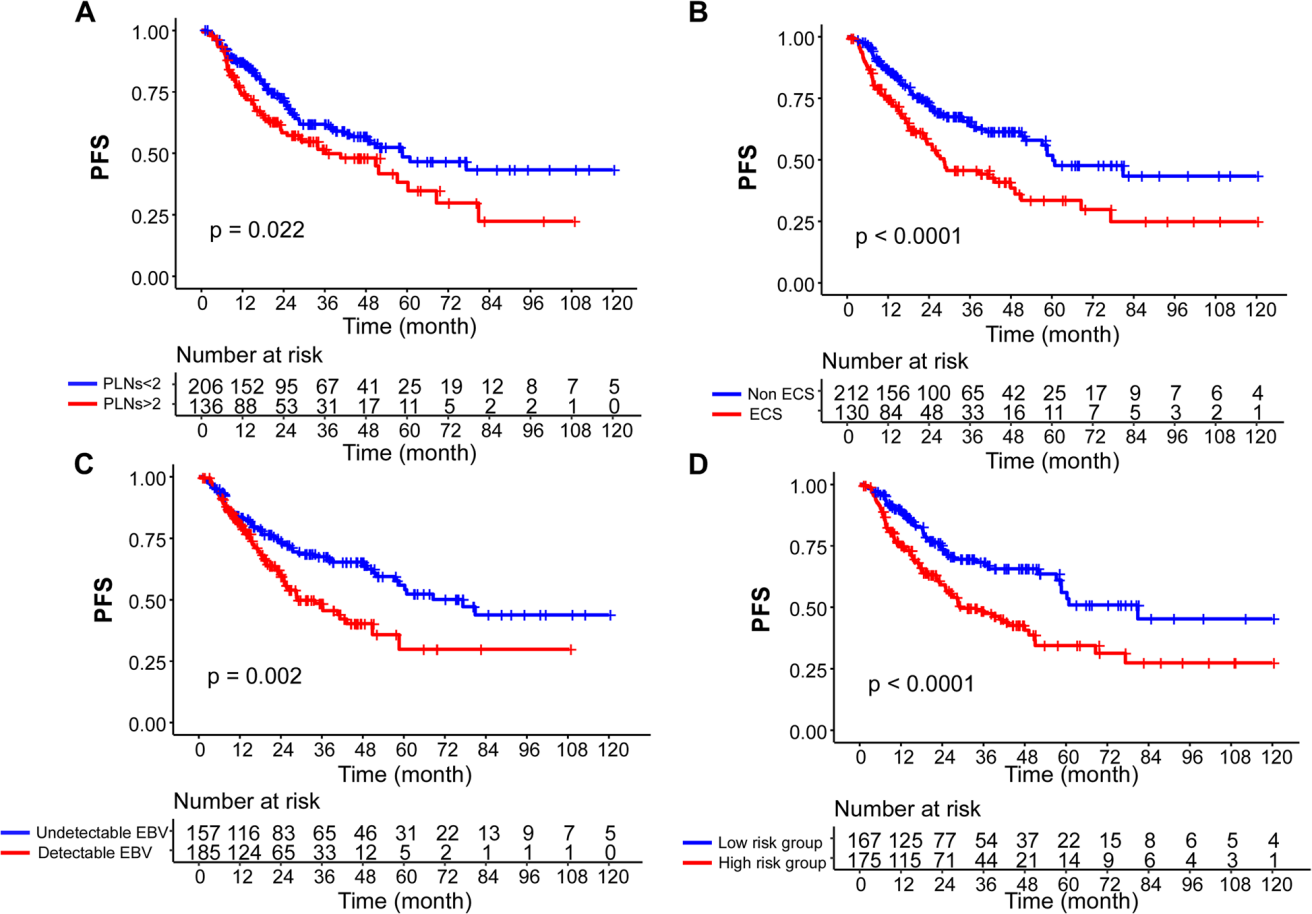
Kaplan–Meier PFS curves in the original cohort. **A** Patients were grouped according to PLNs. **B** Patients were grouped according to ECS. **C** Patients were grouped according to preoperative plasma EBV DNA level. **D** Patients were grouped according to risk stratification

Given that the PLNs (2-year PFS, 71.1% vs. 58.3%, *P* = 0.022), ECS (2-year PFS, 71.9% vs. 56.3%, *P* < 0.0001) and preoperative EBV DNA level (2-year PFS, 73.0% vs. 59.4%, *P* = 0.002) were all independent risk factors for poorer PFS in regional persistent or recurrent NPC patients (Fig. 3A-C). We constructed a prognostic model based on the weight (derived by the b-coefficient of the respective log [AHRs]) of the significant covariates in the whole cohort (Table 2): prognostic score = (0.522 × EBV DNA level) + (0.397 × PLNs) + (0.646 × ECS). The ROC value of prognostic score (0.522) was taken as the cut-off value, and therefore, we divided patients into low-risk and high-risk groups. Patients presenting with ECS or detective preoperative EBV DNA level and PLNs > 2 were classified into high-risk group. The 2-year PFS rates significantly differed among the two groups (73.4% vs. 59.1%, *P* < 0.0001; Fig. 3D).

### The relationship between treatment method and outcomes in different risk groups

**Fig. 4:**
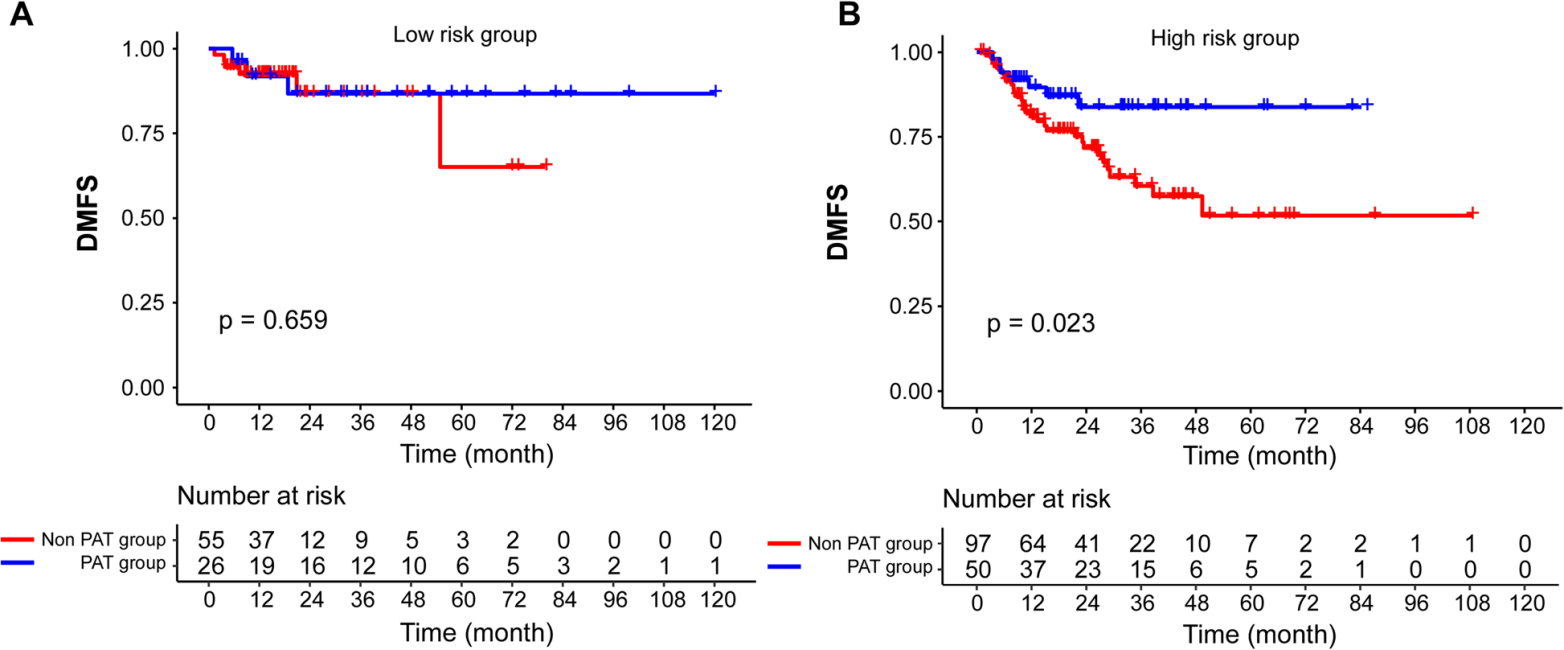
Comparison of DMFS of patients in the ND + PAT and ND alone group. **A** Low-risk patients PSM cohorts. **B** High-risk patients in PSM cohorts

In the low-risk group of the PSM cohort, the addition of PAT failed to bring survival benefits in terms of the 2-year DMFS (86.7% vs. 86.7%, *P* = 0.659, Fig. 4A). However, the 2-year DMFS rate significantly improved with the use of PAT in high-risk group (83.7% vs. 71,7%, *P* = 0.023, Fig. 4B). As for the 2-year PFS, OS and LRFS, there was no significant difference observed between ND alone and ND + PAT groups in all risk groups (data not shown). Table 4 shows that in the high-risk group, a strong prognostic value was indicated for PAT for DMFS (HR 0.616, 95% CI 0.408–0.931, *P* = 0.021). However, PAT did not show significant survival benefits for the low-risk group. In addition, we reanalysed the data after excluding residual neck disease cohort and found that our conclusions still hold true (Supplement Fig. 1).

**Table 4:** Summary of the multivariable analyses of prognostic factors in high risk cohort

**Characteristic**	B	Hazard ratio (95% CI)	*P*
**Progression-free survival**			
Sex	1.015	2.760(1.362–5.594)	0.005
Bilaterality	-1.292	0.275(0.084–0.894)	0.032
ECS	0.725	2.065(1.157–3.683)	0.014
Status of lymph node	0.582	1.789(1.004–3.186)	0.048
**Overall survival**			
Sex	1.464	4.323(1.462–12.780)	0.008
ECS	1.470	4.350(1.830–10.339)	0.001
Status of lymph node	0.900	2.461(1.190–5.088)	0.015
**Loco-regional relapse-free survival**			
preoperative EBV DNA level	0.724	2.062(1.003–4.240)	0.049
ECS	1.024	2.784(1.351–5.735)	0.005
**Distant metastasis-free survival**			
Type of treatment	0.210	0.616(0.408–0.931)	0.021

*Abbreviations*: *CI* confidence interval

HRs and p values were calculated using an adjusted multivariate Cox proportional hazards regression model, Age (y) (> 45 vs. ≤ 45); Sex (M vs. F); rN stage (1–2 vs. 3); EBV DNA (> 0 vs. 0 copy/ml); PLNs (> 2 vs. ≤ 2); ECS (yes vs. no); surgical methods (SND vs. RND); Bilaterality (unilateral vs. bilateral); Status of lymph node (Recurrence vs. Residual); Maximal diameter of LNs (> 20 mm vs. ≤ 20 mm); Type of treatment (surgery with postoperative treatment vs. surgery alone) were included as covariates. Variables were selected with the backward stepwise approach, and the *p* value threshold was 0.1 (*p* > 0.1) for removing insignificant variables from the model. Only variables significantly associated with survival were presented, and marginally significant variables (0.05 < *p* < 0.1) were remained in the final Cox model but not presented in the table

*EBV* Epstein–Barr virus, *PLN* positive lymph node, *ECS* extracapsular spread *RND* radical neck dissection, *SND* selective neck dissection

## Discussions

In this study, to compare the survival outcomes of regional persistent/recurrent NPC patients with or without PAT after ND, we divided patients into high- and low-risk groups based on the prognostic factors, including preoperative EBV DNA level, PLNs and ECS, and found that PAT could significantly reduce the risk of distant metastasis in high-risk patients.

Our study has identified that the PLNs, ECS and the preoperative EBV DNA level closely correlated with PFS for patients with regional persistent/recurrent NPC after neck dissection. The prognostic value of PLNs has been reported by previous studies [16, 17]. We determined the PLN counts based on the postoperative histological results, which was more able to truly reflect the range and severity of invasion than the interpretation from preoperative imaging tests. Although the cut-off value for PLNs varied from 1–3 among previous studies [18, 19], PLNs > 2 was proved by Feng et al. [20] and Li et al. [21] as a reliable cut-off value for risk stratification in head and neck cancers. Extracapsular spread (ECS) is also a well-known poor prognosticator for nodal metastatic NPC patients, which markedly increases the risk of distant metastasis [21, 22]. Our result showed that ECS is an independent prognostic factor for all survival outcomes (PFS, OS, LRFS and DMFS), in keeping with previous studies. The value of EBV DNA as a reliable biomarker for predicting prognosis of NPC has been comprehensively studied before [23–25], and our previous study [26] also demonstrated that for patients with residual nodal disease, the preoperative EBV DNA level was closely related to the prognosis. Therefore, in the present study, we utilized the PLN count, ECS and preoperative EBV DNA levels, and integrated them into risk stratification, which showed good discrimination ability as reflected by the 2-year PFS.

Regarding the treatment strategy, salvage surgery was empirically performed in clinical settings, and studies have been made to discuss about the surgery methods. Wei et al. recommended the use of RND for the high incidence of extracapsular spread observed by their teams [27, 28]. Our results indicated that the surgical method was an independent prognostic factor for DMFS, which was consistent with previous studies. Even though subsequent studies showed that no difference in survival existed between patients receiving RND or SND [29, 30], we believe the neck dissection method should be tailored in selective patients. Nonetheless, high-level evidence from prospective studies are awaited [31]. The next question is whether post-surgery radiation or chemotherapy is necessary for the management of regional residual and recurrent patients. Ji et al. retrospectively analyzed the survival data of residual and recurrent NPC patients, and the 5-year OS and disease-free survival (DFS) were 26.0% and 22.7% respectively, indicating that the prognosis of regional residual and recurrent patients still requires room for improvement. In Zhu et al.’s study, adjuvant chemotherapy failed to improve the DFS and OS of residual patients. Similar conclusions were also drawn by Chan et al.’s study that adjuvant therapy was not associated with survival in residual or recurrent patients [16]. However, the sample sizes of aforementioned studies were small and they did not explore the value of adjuvant therapy according to the different risk stratification.

The application of PAT following ND brought survival benefits to patients in terms of 2-year DMFS in PSM cohort after matching the covariates, such as age, gender, rN stage, etc., and it remained an independent prognostic factor for DMFS in multivariable analysis. We further found that only high-risk group may benefit from the addition of PAT, instead of low-risk patients. For low-risk patients, ND alone group showed similar efficacy as ND + PAT group, which suggested that low-risk patients could be cured by ND alone. As for high-risk patients, the current findings indicated the administration of PAT on these patients is mainly due to the reduction of distant metastasis. It must be noted that high-risk patients in this study were almost presented with ECS. When the extracapsular infiltration of tumor is obvious in imaging tests, the extent of the disease is always extensive under microscope. A prospective study conducted by Chan showed that 80.5% of patients with macroscopic ECS eventually had microscopically involved resection margins even after RND [22]. Therefore, RND alone is not sufficient to entirely eradicate the tumor and more intensive treatment plans should be considered to maximize the therapeutic efficacy, such as postoperative radiotherapy, chemotherapy and immunotherapy. Consistent with our results, Chan’s study showed that satisfactory results could be achieved with combined surgery and brachytherapy in this cohort [22]. Postoperative chemotherapy functions as it kills tumor cells that might have remained following macroscopic tumor removal and eliminated micrometastasis. However, it has not been determined whether combining chemotherapy with RT can improve survival in this salvage therapy setting, and it lacks literature as to which treatment strategy is better for the low incidence of this disease. In this study, no significant difference in clinical outcomes was observed among different PAT methods (data not shown). Consequently, the optimal adjuvant therapy could not be verified. Such observations prompted prospective trials to recommend optimal adjuvant therapy for high-risk patients.

In conclusion, our study developed a risk stratification method for regional residual or recurrent NPC patients based on the PLNs, ECS and the preoperative EBV DNA level, and we found that high-risk patients may benefit from the addition of postoperative adjuvant therapy to neck dissection. Our study has several limitations. Firstly, as this is a single-center retrospective study, the extrapolation of the results needs the validation from external cohorts or prospective trials. Secondly, confined by the sample size, we combined residual and recurrent patients for analysis, but different pathological characteristics may exist among them. Last but not least, the role of post-dissection EBV DNA and its dynamic change should also be explored.

## Conclusions

PLNs, ECS and the preoperative EBV DNA level are associated with the prognosis of patients with regional persistent/recurrent nasopharyngeal carcinoma. In certain cases, high-risk patients identified by PLNs, ECS and preoperative EBV DNA level may benefit from the addition of PAT after neck dissection.

## Supplementary Information

**Additional file 1. **

## Data Availability

The datasets used and/or analysed during the current study are available from the corresponding author on reasonable request.

## Abbreviations

NPC: Nasopharyngeal carcinoma
EBV: Epstein–Barr Virus
IMRT: Intensity-modulated radiotherapy
ND: Neck dissection
PAT: Postoperative adjuvant therapy
RND: Radical neck dissection
SND: Selective neck dissection
rN: Recurrence N
PLNs: Positive lymph node counts
PFS: Progression-free survival
OS: Overall survival
DMFS: Distant metastasis-free survival
LRFS: Locoregional relapse-free survival
DFS: Disease-free survival
MRI: Magnetic resonance imaging
PET/CT: Positron emission tomography/computed tomography
PSM: Propensity score matching
HRs: Hazard ratios
Cis: Confidence intervals
