# Supplementary figures and images for: Identifying optimal candidates for postoperative adjuvant therapy among regional persistent/recurrent nasopharyngeal carcinoma patients after neck dissection
Source: BMC Cancer. 2022 Nov 3;22:1129. doi: 10.1186/s12885-022-10150-0 (PMC9632143; doi:10.1186/s12885-022-10150-0)

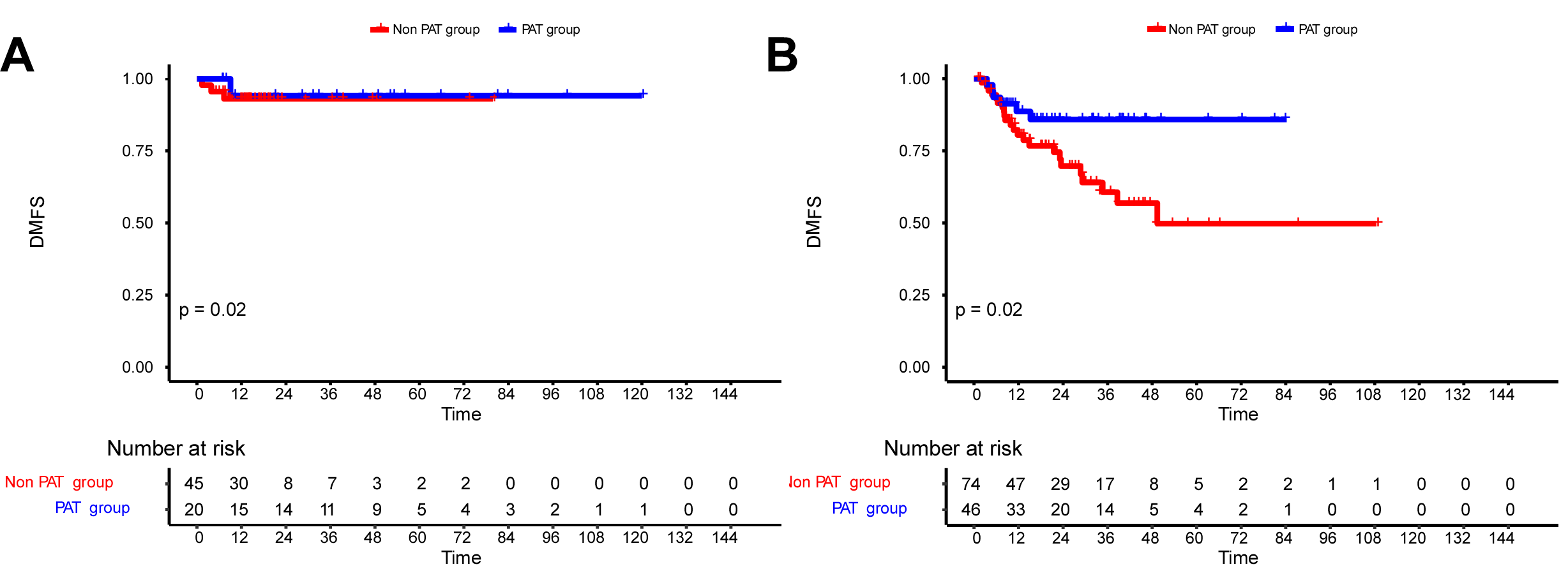

Supplement: Supplementary file 1 — Additional file 1. [file 12885_2022_10150_MOESM1_ESM.tif]
